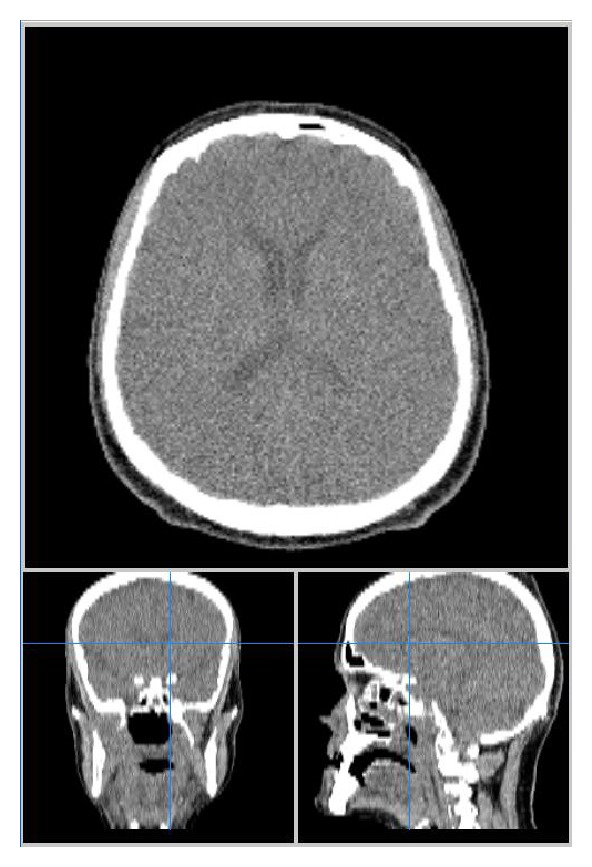# Tension Pneumocephalus Related to Radiotherapy for Nasopharyngeal Carcinoma

**DOI:** 10.1155/2014/327380

**Published:** 2014-08-14

**Authors:** Esther Jimenez-Jimenez, Sebastià Sabater Martí, M. Victoria Villas

**Affiliations:** ^1^Radiation Oncology Department, Hospital Universitari Son Espases de Palma de Mallorca, Palma Health Research Institute (IdIsPa), 07005 Palma de Mallorca, Spain; ^2^Radiation Oncology Department, Complejo Hospitalario Universitario de Albacete, 02006 Albacete, Spain

## Abstract

*Introduction.* Tension pneumocephalus (TP) is a very rare complication related to radiotherapy for nasopharyngeal carcinoma (NPC). *Case Presentation.* A 46-year-old man was admitted to the hospital with an altered mental status and aqueous rhinorrhea for several hours of evolution. The computed tomography (CT) scan showed TP, a defect in the skull base and nasocranial fistula. The patient was receiving a second course of radiotherapy for local relapse. With medical treatment the patient recovered neurological status but died two days later. *Discussion.* In our knowledge, only 4 cases with similar characteristics have been reported in the literature. This is the first case report of TP during radiotherapy. TP was an abrupt and rapid process with neurological impairment for hours of evolution without suspicious osteoradionecrosis (OR) in previous scan images. The defect in the skull base could be due to a rapid disappearance of the tumor. The appearance of aqueous rhinorrhea and neurological symptoms must be viewed as signs of alarm.

## 1. Introduction

Nasopharyngeal carcinoma (NPC) is an uncommon malignant tumor. At diagnosis, the disease is often locally advanced. In these cases, the standard treatment is high doses of conformal radiotherapy with chemotherapy [1].

Pneumocephalus is defined as the presence of gas within any of the intracranial compartments, when a valve mechanism allows air to enter but prevents it to escape a tension pneumocephalus (TP) appears. TP is very uncommon pathology and most cases occur during the postoperative posterior fossa surgery [2]. TP related to radiotherapy is a very rare complication.

We report a case of a patient diagnosed with NPC who developed TP during reirradiation for tumor relapse. In our knowledge, only 4 cases with similar characteristics have been reported in the literature. This is the first case report of TP during radiotherapy.

## 2. Case Presentation

A 46-year-old man was admitted to the hospital with an altered mental status and aqueous rhinorrhea.

Two years earlier, in February 2009, the patient was diagnosed of an undifferentiated NPC Stage IVA (cT4N2M0) according to AJCC's 7th edition manual, with involvement of cranial nerves and orbit. He was treated with induction cisplatin-5-fluorouracil scheme, for two cycles followed, from May 2009 to June 2009, by concurrent chemoradiotherapy with cisplatin.

Gross tumor volume (GTV) was determined based on magnetic resonance imaging (MRI) and computed tomography (CT) tumor extent. Clinical target volumes (CTV) were GTV + 1 cm and nodal levels at-risk (bilateral nodal level IB, II, III, IV, V, and retropharyngeal). Planning target volumes (PTV) were CTV plus a 0.5 cm margin. Radiation doses were 70 Gy to tumor and involved nodes and 50 Gy to prophylactic anatomic sites. Fractionation was 2 Gy/session. The treatment was planned using 6 MV and 15 MV photon energy. Usual head-and-neck critical structures, such as spinal cord, were contoured.

Patient remained in complete remission, documented with endoscopy and MRI, up to May 2010 when a local recurrence was diagnosed. A CT scan showed a tumor of 3 × 2 × 1.5 cm^3^ with intracranial extension and involvement of skull base. Five cycles of cisplatin-5-fluorouracil were scheduled. At the end stable disease was evident on CT. No complaints about neurological or other type of symptoms were reported.

In March 2011, the patient complained of mild pain in left eye, moderate periorbital swelling, and ophthalmoplegia without other neurological symptoms. On MRI local progression appeared, with tumor invasion of the pterygoid and masseter muscle in the temporal fossa, sphenoid sinus, ethmoid cells, orbit apex, and meningeal involvement in ipsilateral temporal fossa. Osteoradionecrosis (OR) was not described by the radiologist.

At that time, a reirradiation of the visible tumor plus 1 cm margin with an imaged-guided intensity-modulated radiotherapy (IMRT) technique, in order to decrease the toxic effects of reirradiation, was decided. CTV was GTV + 1 cm and a total dose of 66 Gy was planned. Reirradiation began in April 2011 without any neurological symptom. Treatment was stopped at 64 Gy because he exhibited abrupt deterioration.

Patient was admitted to the hospital because of incoherent speech, impaired behavior, and aqueous rhinorrhea for several hours of evolution. In the emergency room, physical examination revealed disorientation as to time and place, cerebrospinal fluid rhinorrhea, left periorbital swelling, and fever (38.3°). Neurological examination confirmed a Glasgow coma scale of 10 and cranial nerves could not be properly assessed. Other findings were unremarkable. Culture of the ventricular fluid was not realized.

**Figure 1:**
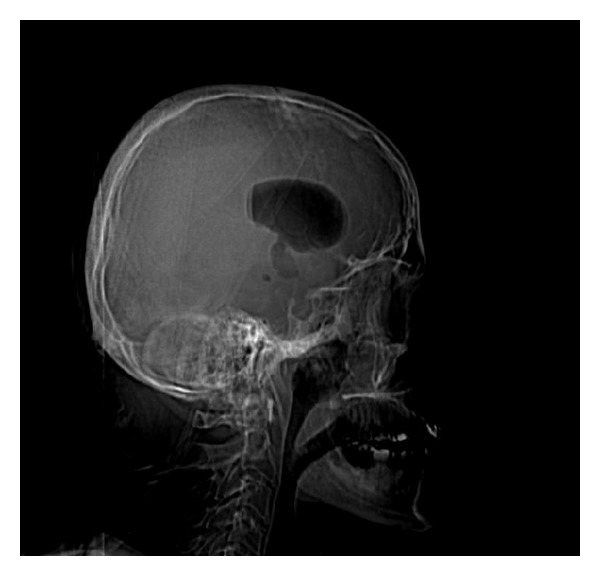


**Figure 2:**
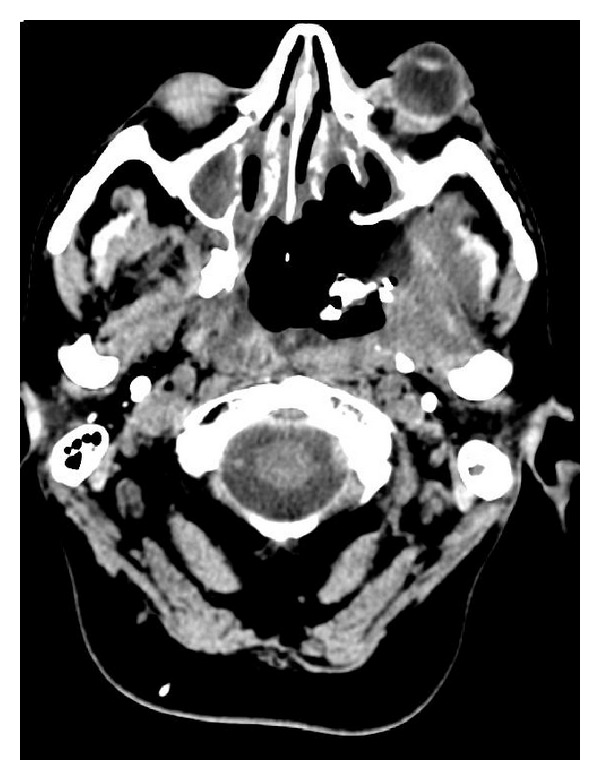


**Figure 3:**
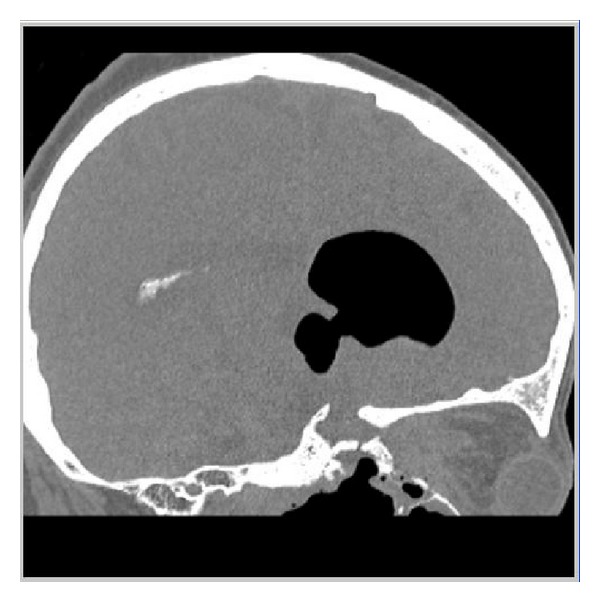


**Figure 4:**
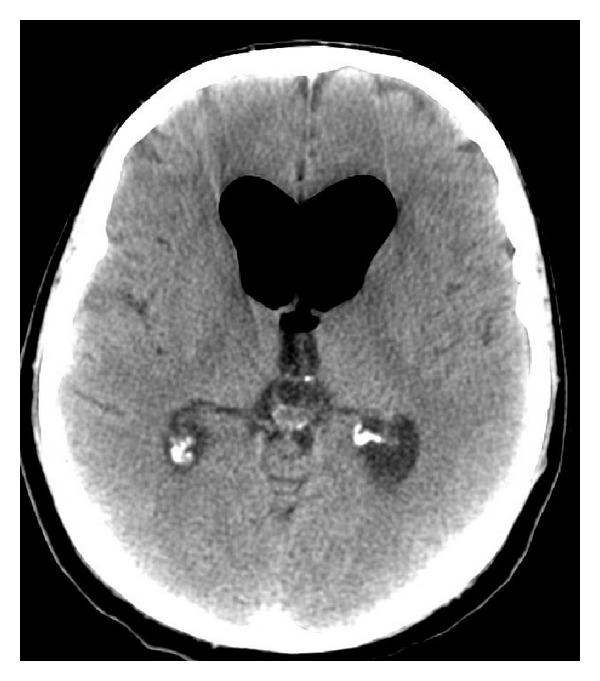


**Figure 5:**
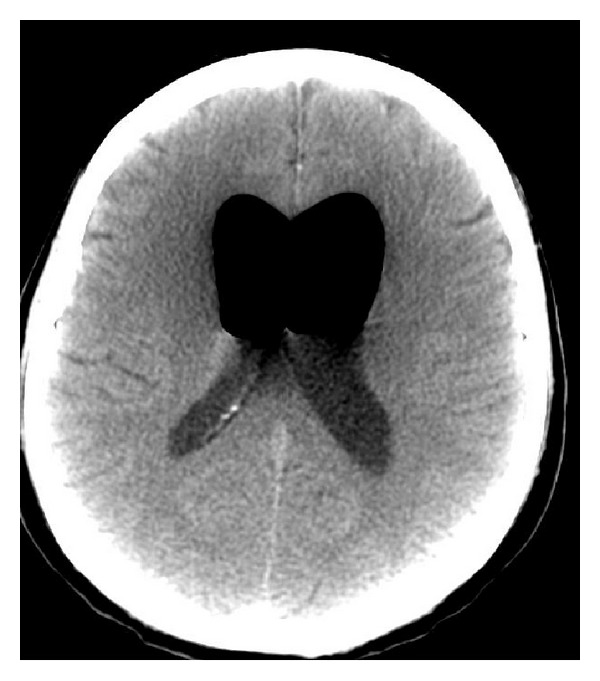


Radiography showed the presence of cranial air in the lateral and III ventricles (Figure 1). The base of the skull appeared broken (Figure 2). A linear fracture was detected in the left side using bone window settings. A brain CT scan revealed a TP (Figures 3 and 4) and intraventricular gas was noted within symmetrically dilated frontal horns of the lateral ventricles (Figure 5). Two weeks before, these findings were not evident on the planning CT (Figure 6).

Patient's poor clinical situation and the high local tumor invasion impeded aggressive treatments. He was treated with intravenous fluid, broad spectrum antibiotics, and anti-inflammatory drugs. With medical treatment the patient recovered neurological status, but the patient died two days later.

## 3. Discussion

Pneumocephalus refers to the presence of intracranial gas and is a very rare complication of radiotherapy in head and neck. It is usually caused by several etiologies, such as craniofacial surgery, trauma, tumors, or meningitis from gas forming organisms. TP associated with radiotherapy is an exceptionally rare and potentially fatal complication. The majority of cases of pneumocephalus are asymptomatic, but in TP the mass effect can produce related symptoms. Treatment depends on cause. The majority of postcraniotomy cases with no treatment is necessary with the air being reabsorbed. Surgery is necessary in the presence of TP in order to relieve pressure or in cases of cerebrospinal fluid leakage.

Generally, only minor complications are observed as a result of a single course of radiotherapy for NPC: xerostomia, trimus, fibrosis, and so forth. However, severe complications can result in patients subjected to second salvage radiation, such as myelitis, paralysis of nerves, OR, temporal lobe injury, and severe trimus [3].

Our review of the literature revealed 6 cases of pneumocephalus related to NPC and only 4 cases of TP. Ng et al. [4] are the first to report a case of TP in association with NPC. The patient was a 60-year-old male diagnosed of locally advanced NPC. He received 64 Gy in 32 fractions and almost 4 years later was admitted in hospital with TP. This author reported that TP was due to a nasocranial fistula in an area of OR that showed in postmortem findings. CSF rhinorrhea was not documented and the base of the skull appeared sclerotic. The patient died about 5 weeks after.

Kiu et al. [5] presented a 55-year-old man with recurrent NPC, treated with radiotherapy (8380, cGy). The patient was admitted with TP. CT revealed OR breaking through the skull base. He was treated with a burr hole but finally died due to fulminant meningitis.

Wu and Lee [6] reported a 59-year-old man, diagnosed as NPC Stage 2, treated with radiotherapy (68 Gy). Three years later, the tumor relapsed and he received salvage radiotherapy (50 Gy) with chemotherapy (cisplatin+5-fluorouracil). One year after, an open biopsy of the temporal lobe was performed for a lesion. The pathological examination revealed radionecrosis, but not tumor. Two years later a cranial CT disclosed TP. This patient was repaired the fistula. The pathological examination exposed radionecrosis of the frontal skull base and no tumor cells were found. He was discharged 2 weeks after surgery.

Wang et al. [7] presented a 45-year-old man diagnosed with NPC Stage III. A course of radiotherapy (70 Gy/39 fractions) was administered. Two years later the patient suffered from intermittent clear fluid from the nose. Next year he was brought to the hospital with TP. The patient underwent an emergency intraventricular drainage tube insertion for decompression and then he received a craniotomy to repair the defect of the right skull base. The patient recovered well. The bone biopsy from the right skull base showed extensive bone necrosis.

In the reported cases, TP appeared between 2 and 4 years after radiotherapy. All cases detailed the presence of OR as the cause of the skull base defect. As in previous reports, in this case TP appeared 2 years after the first radiotherapy course.

Wang et al. presented a subacute case, with rhinorrhea months before TP. In the other cases, the presence of rhinorrhea was not documented. In our case, the patient who was in radiotherapy treatment did not complain about rhinorrhea, dizziness, headache, or other neurological symptoms. The clinical onset was abrupt with symptoms and neurological impairment for several hours of evolution.

We believe that our patient suffered from an OR in the skull base. This event usually occurs 1 year after radiotherapy. It is thought to be secondary to osteoblastic destruction. The skull base, cervical spine, and the mandible are commonly affected. Imaging findings include areas of osteolysis and mixed sclerosis [3]. If an OR of the skull base occurs, air may be introduced into the cranial cavity.

However, patient presented an abrupt and rapid TP so another possibility could be the creation of a defect in the skull base due to a rapid tumor disappearance, which previously invaded the skull base. In addition, signs of OR in MRI or CT were not described by the radiologist. The main drawback of our work is the lack of autopsy, precluding verify if tumor disappearance and/or OR were the responsible of the TP. The autopsy would confirm or refuse our hypothesis.

Despite high-dose reirradiation being difficult due to the previous doses to the nearby critical structures, IMRT allows achieving better dose distributions than previous techniques, so doses higher than 50 Gy can be delivered for salvage irradiation. Doses higher than 60 Gy are associated with better outcomes [8–10].

The principles of treatment of TP are to provide adequate supportive care and antimicrobial therapy and to modify the hot's inflammatory response [11]. In addition, TP must be decompressed rapidly, and it is usually treated with surgical decompression and correction of the underlying cause. In our patient, the poor clinical situation and the local tumor invasion on the last MRI impeded aggressive treatments.

## 4. Conclusions

Radiotherapy is a very useful arm in the treatment of NPC, in primary or salvage treatment. High-dose irradiation is necessary to attempt curative treatments, and serious complications have been described.

TP is a very rare complication of radiotherapy related to NPC, but this possibility must be kept in mind when treating primary cancer patients with tumors invading the skull base, with neurological symptoms or when planning a salvage reirradiation. Patients complaining about neurological symptoms or persistent rhinorrhea must been viewed as signs of alarm. Rapid diagnosis of TP could be vital for the patient.

## Figures and Tables

**Figure 6:**